# The Community Structure of Aerobic Anoxygenic Photosynthetic Bacteria in Biocrusts on Tropical Coral Islands and Their Application in Ecological Restoration, South China Sea

**DOI:** 10.3390/microorganisms13061265

**Published:** 2025-05-29

**Authors:** Jing Wen, Zhimao Mai, Jie Li, Lin Wang, Si Zhang

**Affiliations:** 1 CAS Key Laboratory of Tropical Marine Bio-Resources and Ecology, South China Sea Institute of Oceanology Chinese Academy of Sciences, Guangzhou 510301, China; wenjing23@mails.ucas.ac.cn (J.W.); maizhimao@scsio.ac.cn (Z.M.); lijietaren@scsio.ac.cn (J.L.); 2 University of Chinese Academy of Sciences, Beijing 100049, China

**Keywords:** tropical coral island, biocrust, aerobic anoxygenic phototrophic bacteria, diversity, ecological restoration, environmental driver factors

## Abstract

Biological soil crusts (referred to as biocrusts) constitute prominent components within the ecosystem of tropical coral islands in the South China Sea, covering approximately 6.25% of the island’s terrestrial surface. Biocrusts are the key to the restoration of the island ecosystem. It is widely acknowledged that phototrophic microorganisms profoundly contribute to biocrust formation and development. They provide fixed carbon and nitrogen and produce exopolysaccharides for the BSC ecosystems. Although aerobic anoxygenic phototrophic bacteria (AAPB) are an important functional group of phototrophic microorganisms, the community characteristics of AAPB in coral island biocrusts and their role in the formation of biocrusts have rarely been reported. In this study, we employed amplifications of the *puf*M gene to characterize the AAPB communities of biocrusts on a tropical coral island. The outcomes revealed a discernible augmentation in both the abundance and richness of AAPB concurrent with the formation of biocrusts, concomitantly with a decrement in diversity. Within the AAPB communities, the *Pseudomonadota* (*Proteobacteria*) phylum emerges as the prevailing dominion, indicating marked differentiations in terms of family and genus compositions between the biocrust and bare soil. Canonical correlation analysis has unveiled a robust and meaningful correlation between the AAPB composition and the attributes of the soil, including total nitrogen, total organic carbon, total phosphorus, pH, and calcium content. Furthermore, co-occurrence network patterns shift with biocrust formation, enhancing stability. Meanwhile, keystone taxa analysis revealed specific OTUs associated with each soil type, with genus *Brevundimonas* as the main group. Furthermore, pure-culture AAPB strains isolated from biocrusts exhibited a panorama of diversity, predominantly belonging to *Pseudomonadota*. Particularly, the *Skermanella* and *Erythrobacter* genera demonstrated strong exopolysaccharide secretion and sand-binding capabilities. This study sheds light on the significant functional role of AAPB in tropical coral island biocrusts, expanding our understanding of their contribution to ecosystem services, and providing valuable insights for ecological restoration efforts on coral islands.

## 1. Introduction

Tropical coral islands play a crucial role in upholding the biodiversity of marine ecosystems, serving as crucial waystations for migratory avian species and marine mammals. Additionally, they have made significant contributions to the preservation of freshwater reserves and local climate regulation [1,2,3]. Nevertheless, due to the predominant composition of these coral islands being coral calcareous sands, with calcium carbonate content reaching levels as high as 90%, they are not conducive to the natural colonization of vegetation. Moreover, in terms of both the environment and biota, these coral islands are deemed as “deserts” within the marine ecosystem, marked by an absence of authentic soil substrates and freshwater reservoirs. They exhibit characteristics of extreme environments, including high salinity, intense alkalinity, elevated temperatures, and intense luminosity. Concomitantly, severe soil erosion is observed. These adverse conditions are counterproductive to the ecological functionality of coral islands. Thus, the pivotal undertaking of improving and restoring the ecosystem of coral islands is instrumental in steering these “desert” islands from desolation to thriving ecological habitability. In this endeavor, biological soil crusts (biocrusts), highly esteemed in arid ecosystems of analogous extremity, offer profound inspiration for reshaping the “desert” state of these islands.

Biocrusts formed by microorganisms, algae, mosses, lichens, and other organisms in conjunction with soil exhibit characteristics such as drought resistance, salinity tolerance, carbon and nitrogen sequestration capabilities, and robust reproductive capacities [4,5]. They thrive and proliferate extensively in harsh environments, influencing their immediate surroundings through physiological and metabolic processes [6]. Encompassing 12% of the Earth’s terrestrial surface [7], biocrusts emerge as substantial constituents of terrestrial ecosystems. In regions marked by nutritional scarcity, these biocrusts act as pioneers of nature, enhancing soil stability, curbing nutrient runoff, ameliorating soil microenvironments, and heightening soil water retention and runoff delay [8,9]. Additionally, they fulfill pivotal ecological roles encompassing erosion resistance, carbon fixation, and nitrogen fixation, thus laying the groundwork for nutritional elements and a soil microenvironment conducive to the robust growth of ecosystems [10,11,12]. Within the developmental journey of biocrusts, photoautotrophic organisms play a crucial role [4,5,13,14]. Among them, cyanobacteria emerge as crucial participants in biocrust development, contributing by atmospheric carbon and nitrogen fixation and secreting polysaccharides that agglomerate sand particles, augmenting soil nutrient content [5,13,15]. Yet, aerobic anoxygenic photosynthetic bacteria (AAPB), sharing analogous functional attributes, have remained in the shadows concerning their role in the biocrusts. Scant reports delve into their community structure, diversity, and impact on biocrust formation and development [16,17].

AAPB are a group of bacteria capable of producing bacteriochlorophyll a (BChl *a*) and engaging in photosynthesis mediated by BChl *a* under aerobic conditions yet not generating oxygen [16]. AAPB represent the second-largest branch of photosynthetic organisms on Earth [18], constituting around 20% or more of the aerobic microbial community [19]. AAPB have been discovered to abound in terrestrial biocrust habitats, including forests [20], high-altitude wastelands [14], and desert fringes [21], indicating their vital role within biocrust ecosystems. AAPB employ photosynthesis to generate ATP, supplementing energy required for growth, thus curbing the consumption of organic carbon in the environment [22]. In comparison to heterotrophic organisms reliant solely on organic carbon for growth, AAPB exhibit heightened competitiveness [17]. Moreover, AAPB increase the influx of dissolved organic matter into cells, thus contributing significantly to biogeochemical cycles [17]. Research by Tang et al. [21] indicated that within biocrust ecosystems, AAPB served as a critical functional group, fostering the rapid development of biocrusts and closely correlating with biocrust succession stages and soil organic carbon content. Due to the potentially comprehensive genomes of AAPB, which may include genes for extracellular polysaccharide production, N_2_-fixation, and CO_2_ sequestration [13,21], existing studies have confirmed these capabilities in certain AAPB species, including atmospheric nitrogen fixation [23], carbon dioxide fixation [24], and extracellular polysaccharide synthesis [25]. Previous research underscores the affirmative role of microbes and their secreted polysaccharides in biocrust formation [26]. In the early stages of biocrust formation, extracellular polysaccharides (EPS) secreted during microbial metabolism bind soil particles, creating aggregates that enhance soil stability, reduce erosion, and improve nutrient retention [27,28,29,30]. Evidently, EPS-secreting AAPB play a pivotal role in biocrust formation. Additionally, preliminary research suggests that cyanobacteria utilize visible red light with wavelengths between 514 and 700 nm, whereas AAPB exploit near-infrared light with wavelengths between 760 and 1130 nm [31]. The two bacterial groups complement each other in light utilization, theoretically maximizing the biocrust’s utilization of solar energy. Furthermore, given the proximity of coral islands to the equator and their reduced greenhouse gas concentrations and cloud cover, the intensity of near-infrared light in this region is substantially higher than in other areas, rendering the biocrust habitat suitable for AAPB survival. Simultaneously, in the nutrient-poor environment of island terrestrial regions, AAPB can utilize their competitive advantage for development and reproduction, thereby influencing carbon and energy cycling within the island ecosystem. This suggests that AAPB might serve as a significant functional component in biocrusts on the tropical coral islands of the South China Sea, contributing significantly to biocrust formation, development, and critical ecosystem services. Nevertheless, our understanding of the community characteristics of such microorganisms in coral islands and their environmental influencing factors remains limited [16,17].

Here, our aim is to address the following questions: (1) How does the community structure of AAPB in coral island biocrusts vary, and what environmental factors are associated with it? (2) Do pure cultures of AAPB possess the potential to secrete extracellular polysaccharides and enhance soil particle aggregation under laboratory conditions? In this study, we intend to investigate the aforementioned scientific inquiries through a combination of high-throughput amplicon sequencing methods and laboratory-simulated cultivation techniques. The outcomes of this research are anticipated not only to comprehensively elucidate the community characteristics of AAPB within the coral islands and their environmental influencing factors but also to potentially impact the enhancement of coral island biocrusts and the restoration of island ecosystems.

## 2. Materials and Methods

### 2.1. Sampling Area

The samples, including biocrust and bare soil, were collected from a coral island in the South China Sea during September 2021. The coral island is located in the tropical low-latitude maritime monsoon climate zone [32] and experiences the influence of a tropical marine climate, characterized by an average temperature exceeding 27 °C and an annual precipitation surpassing 2000 mm. Furthermore, it experiences strong maritime winds throughout the year [33]. The predominant soil composition on the coral island is coral calcareous sand, predominantly composed of CaCO_3_, exhibiting irregular shapes, porous characteristics, fragility, and a relatively high angle of friction [34,35]. In open coral calcareous sand areas, the terrain is wide, flat, and sparsely vegetated, primarily dominated by species such as *Eleusine indica*, *Fimbristylis cymosa*, and *Cyperus rotundus*. The coverage of biocrusts on unvegetated surfaces is approximately 6.25% and predominantly consists of cyanobacterium-crust [34].

### 2.2. Sampling and Storage

In this study, we collected two types of samples (no plants within 2 m): (1) biocrust samples (0–1 cm of soil surface); (2) bare soil (0–1 cm of soil surface of soils without biocrusts). All of the samples, including biocrusts and bare soil, were at least 50 m apart and collected using sterile spatulas. In this study, we collected 12 soil samples from the coral island, South China Sea: 6 biocrust and 6 bare soil samples.

During the sample collection process, three copies of each sample were collected: one for the measurement of soil physicochemical properties, which was kept in a −20 °C refrigerator; one for qPCR and high-throughput sequencing analysis, which was initially preserved in liquid nitrogen, brought back to the laboratory immediately, and stored at −80 °C until the further experimental requisites; the last one was used for the isolation and purification of AAPB strains, and the storage temperature was 4 °C.

### 2.3. Measurement of the Soil Physicochemical Properties

Firstly, all samples were air-dried at 25 °C. Then, the coarse impurities were removed using a 35-mesh screen, and the remaining samples were ground with a mortar and pestle. Soil pH value was analyzed with a pH meter (pH 211, Hanna Instruments, Vöhringen, Germany) [36]. Total organic carbon (TOC) and total nitrogen (TN) were measured by the Elementary Analyzer (Flash EA 3000 Thermo Scientific, Milan, Italy) [37]. Total phosphorus (TP) and calcium (Ca) content were calculated based on methods described by Wang et al. [34].

### 2.4. Isolation, Purification, Identification, and Functional Screening of AAPB

This section describes a culture-dependent approach to obtain and functionally characterize AAPB strains isolated from biocrust and bare soil samples. The collected samples were subjected to thorough pulverization, vigorous oscillation, and gradient dilution. Subsequently, the diluted samples were individually spread onto R2A, TSA, MA, and BG11 culture media (Hopebio, Qingdao, China) and cultured at 27 °C for 2–4 days. Colonies were selected based on their morphologies, picked from the plates, and further purified using streaking on fresh culture media.

A bacterial genome DNA extraction kit (DP302, Tiangen, Beijing, China) was employed to extract bacterial genomic DNA. Meanwhile, the universal bacterial primers 27F (AGAGTTTGATCCTGGCTCAG) and 1492R (GGTTACCTTGTTACGACTT) were employed to amplify the bacterial 16S rRNA gene. The AAPB biomarker gene *puf*LM was amplified using primers 67F (TTCGACTTYTGGRTNGGNCC) and 781R (CCAKSGTCCAGCGCCAGAANA) and then sequenced. The 16S rRNA gene sequences obtained from Sanger sequencing were compared online through the EzBioCloud Website to determine the closely related species of the isolated strain. According to the results of the 16S rRNA gene sequence alignment, the 16S rRNA gene sequence of the isolated strain with the highest similarity was used as a reference, and the Clustal W method in the MEGA-X software was used for multiple sequence alignment [38]. The aligned results underwent model prediction, with the best-fit model being Tamura–Nei (TN93). The neighbor-joining method was employed for clustering analysis to construct a phylogenetic tree [39]. The tree was bootstrapped 1000 times, and visualization was enhanced using the iTOL website [40].

### 2.5. DNA Extraction, Amplification, and Sequencing

In contrast, this section presents a culture-independent method based on high-throughput sequencing of the *puf*M gene to analyze the community structure of AAPB directly from environmental DNA. We employed the DNeasy^®^ PowerSoil^®^ Pro Kit (QIAGEN, Germantown, MD, USA) for the extraction of total environmental DNA, following the instructions provided in the accompanying manual. For the amplification of the *puf*M gene, a specific primer set targeting AAPB was utilized, namely *puf*M557F (TACGGSAACCTGTWCTAC) and *puf*MWAWR (CCATSGTCCAGCGCCAGAA) [14]. Detailed polymerase chain reaction conditions for the *puf*M gene could be referenced from the work of Yang and Hu [14]. Subsequent sequencing was executed on the Illumina PE300 platform (Meiji Biomedical Technology Co., Ltd., Shanghai, China). The raw sequencing data have been deposited in the National Center for Biotechnology Information (Bethesda, MD, USA) (Study accession number PRJNA1003425).

The raw paired-end sequencing reads underwent quality control utilizing the fastp software (v0.19.6) [41] and were merged using the FLASH software (v1.2.11) [42]. The quality-controlled and merged sequences were then processed using the UPARSE v7.1 software [43]. Operational taxonomic units (OTUs) were generated through clustering the quality-controlled and merged sequences at a 97% similarity threshold, with the removal of chimeric sequences. Taxonomic annotations for the OTUs were assigned using the RDP classifier [44] with the NT database (v20210917) as the reference, applying a confidence threshold of 70%. The composition of the microbial communities in each sample was ascertained at various taxonomic levels.

### 2.6. Real-Time PCR

To quantify the cell abundance of AAPB, the maker gene copies of *puf*M gene were measured by qPCR. The primer pairs of *puf*M557F and *puf*MWAWR were used. Precise quantification was conducted utilizing the LightCycler 480 System (Roche, Basel, Switzerland). Standard curves were constructed through progressive dilution of plasmids containing the marker gene, resulting in ultimate concentrations ranging from 103 to 108 copies/µL. Triple qPCRs were executed across all samples and concentrations outlined in the standard curve. Amplification specificity was validated through a meticulous analysis of melting curves, followed by subsequent electrophoresis in a medium of 2% agarose gel. The copy number of the marker gene was expressed as per gram of wet weight in the soil samples.

### 2.7. Statistical Analyses

QIIME v 1.8.0 [45] was applied to calculate the α-diversity values (e.g., Sob index, Ace index, and Shannon index) of microbial community. A *t*-test was conducted to assess the significance of the differences between biocrusts and bare soil regarding soil properties, *puf*M gene copies, bacterial relative abundance, and the values of α-diversity by using SPSS v18. Permutational multivariate analysis of variance determined the statistical significance of the differences between biocrusts and bare soil (Vegan v2.5–3 in R) [46]. The canonical correlation analysis (CCA) investigated the impact of soil properties on the AAPB composition (Vegan v2.5–3 package in R). A co-occurrence correlation of AAPB in different types of samples was calculated by applying the R software’s psych package v1.8.12 [47]. The co-occurrence networks were visualized using Gephi v0.9.2 [48]. The topological characteristics of co-occurrence networks were calculated, and the robustness of networks was analyzed by random node removal using natural connectivity [49,50].

## 3. Results

### 3.1. Physicochemical Soil Properties

The study findings indicated that the total nitrogen (TN), total phosphorus (TP), and total organic carbon (TOC) content in bare soil were significantly lower compared to biocrusts (*p* < 0.05, Table S1). Notably, the content of TOC, TN, and TP in the biocrust could reach up to 20.25 g/kg, 2.06 g/kg, and 1.59 g/kg, respectively. Ca content and soil pH values were notably higher in bare soil compared to the biocrust, implying that the formation of biocrust could lead to a reduction in Ca content and pH values.

### 3.2. Culture-Independent Analysis of AAPB Community Diversity and Composition

**Figure 1:**
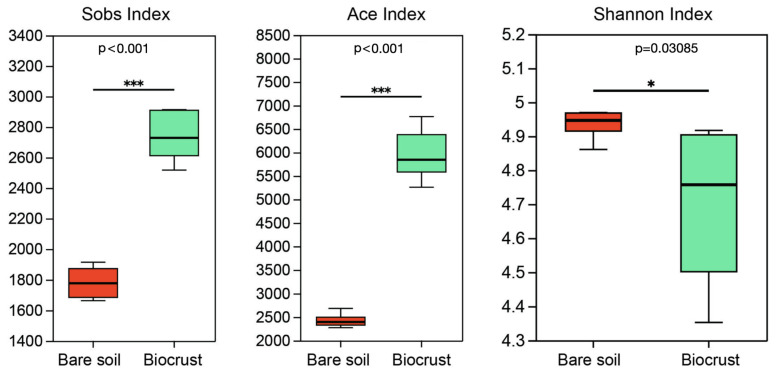
Comparison of richness and diversity of AAPB community in the biocrust and bare soil samples. The boxplot demonstrates the α-diversity parameters, Sobs index, Ace index, and Shannon index. Boxes limit the 25th and 75th percentile with the median presented as line inside. Error bars present the 1st and 99th percentile. Significant differences: *** *p* ˂ 0.001, * *p* ˂ 0.05.

Regarding the α-diversity of AAPB, we employed the Sobs index to characterize microbial abundance, the Ace index to represent species richness, and the Shannon index to delineate the species diversity of AAPB (Figure 1). The research findings demonstrate that the abundance and species richness of AAPB in the biocrust were significantly higher than those in bare soil (*p* < 0.05). This outcome was consistent with the qPCR results, which showed that the studied gene copy was significantly higher in biocrusts than in bare soil (Table S2). However, the species diversity of AAPB in the biocrust was significantly lower compared to bare soil (Figure 1, *p* < 0.05).

**Figure 2:**
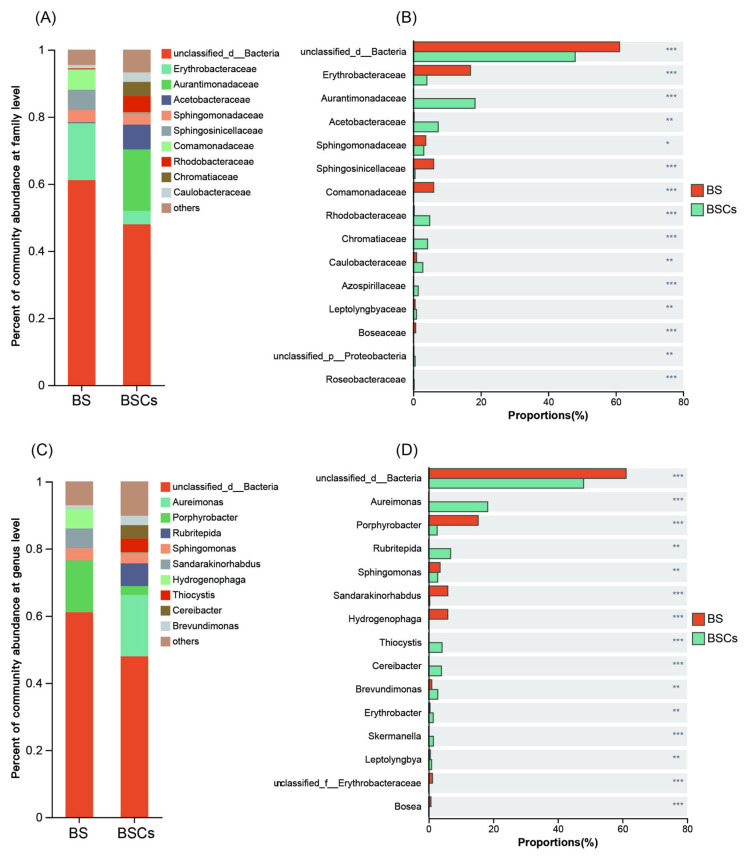
Analysis of differences of microbial composition between the bare soil and biocrust. (**A**) Relative abundance of the major AAPB taxa at family level; (**B**) difference analysis of dominant taxa at family level; (**C**) relative abundance of the major AAPB taxa at genus level; (**D**) difference analysis of dominant taxa at genus level. Based on amplicon 16S rRNA gene data. Significant differences: *** *p* < 0.001, ** *p* < 0.01, * *p* < 0.05. Abbreviations: bare soil (BS), biocrusts (BSCs), and aerobic anoxygenic photosynthetic bacteria (AAPB).

For AAPB composition, the *Pseudomonadota* phylum was dominant in both bare soil and biocrust. At the family level, the major taxa in the biocrust samples were *Aurantimonadaceae* (average: 18.3%), *Acetobacteraceae* (7.4%), and *Rhodobacteraceae* (4.9%), significantly higher than in bare soil (Figure 2A,B). For bare soil samples, the most abundant family was *Erythrobacteraceae* (16.9%), with smaller contributions of *Sphinggosinicellaceae* (6.1%) and *Comamonadaceae* (6.0%). At the genus level, the predominant taxa in the biocrusts were *Aureimonas* (18.3%), *Rubritepida* (6.8%), and *Thiocystis* (4.2%), significantly higher than in the bare soil (Figure 2C,D). Furthermore, *Porphyrobacter* (15.3%), *Sandarakinorhabdus* (5.9%), and *Hydrogenophaga* (5.9%) were significantly higher in the bare soil (Figure 2D).

In both the biocrust and bare soil samples, a proportion of AAPB OTUs remained unclassified at the family or genus level (Figure 2A,C). These “unclassified” taxa likely represent poorly characterized or novel AAPB lineages that are underrepresented in current reference databases. The presence of unclassified OTUs, particularly in bare soil samples, may reflect a unique AAPB subcommunity adapted to nutrient-poor or early-successional environments. Future efforts involving full-length 16S rRNA or metagenomic sequencing could help resolve the taxonomy and potential functions of these unclassified members. To provide a comprehensive visual overview of the microbial diversity in both biocrust and bare soil samples, we constructed a phylogenetic tree based on species-level taxonomy of all detected OTUs (Figure S1). This tree illustrates the overall phylogenetic relationships among taxa and highlights their distribution across sample types, with node colors indicating whether each species was identified in biocrust, bare soil, or both environments.

**Figure 3:**
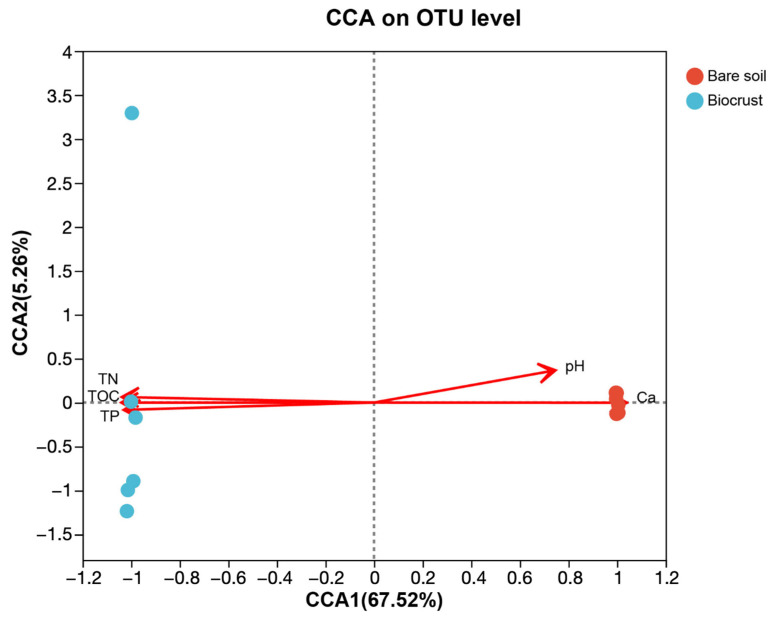
The canonical correlation analysis of AAPB community structure and its correlation with environmental factors. The circles represent soil samples; different soil types are color-coded. Abbreviations: total nitrogen (TN), total phosphorus (TP), total organic carbon (TOC), calcium content (Ca), and aerobic anoxygenic photosynthetic bacteria (AAPB).

Differential analysis of AAPB community composition among various sample groups was conducted using PERMANOVA. We discovered significant differences in the composition of AAPB communities between the biocrusts and the bare soil. Through canonical correlation analysis, we found that AAPB composition was highly correlated to the soil properties, including TOC, TN, TP, pH, and Ca contents (Figure 3).

### 3.3. The Co-Occurrence Network of AAPB

**Figure 4:**
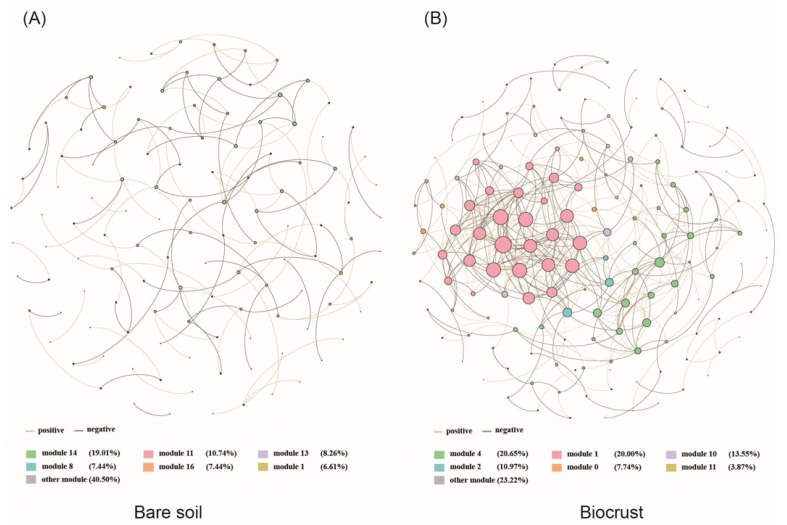
Co-occurrence network of AAPB communities in (**A**) bare soil and (**B**) biocrust samples. Each node represents an OTU; edge thickness indicates correlation strength. Node size is proportional to degree (number of connections), and node colors indicate topological modules identified using the modularity.igraph function from the R igraph v2.1.2 package.

Our results indicated that AAPB community co-occurrence network patterns clearly shifted with biocrust formation (Figure 4). The network connectivity, indicated by average degree, was higher in the biocrust (5.95) compared to that in the bare soil (1.09) (Table S3). The network complexity, indicated by number of edges, also showed the same pattern of the network connectivity (Table S3). Through analysis of Zi and Pi, we found that OTU11998 (unclassified) was considered a keystone OTU for bare soil. In addition, OTU5916 (*Brevundimonas*), OTU6054 (*Thiocystis*), OTU5691 (*Sphingomonas*), OTU6025 (unclassified), OTU6288 (*Brevundimonas*), and OTU6192 (unclassified) were keystone OTUs for biocrusts (Table S4). The network analysis documented that the AAPB community enhanced stability with biocrust formation on the coral island.

### 3.4. Culture-Dependent Isolation and Taxonomic Diversity of AAPB Strains

**Figure 5:**
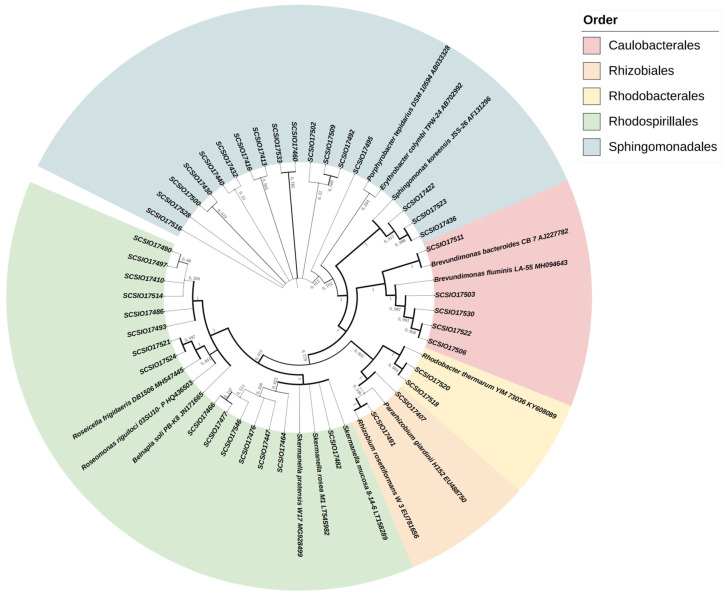
Phylogenetic tree of AAPB strains constructed by the neighbor-joining method based on 16S rRNA gene sequences. Abbreviations: aerobic anoxygenic photosynthetic bacteria (AAPB).

Through isolation and purification, we successfully obtained and identified 246 bacterial strains, of which 41 were identified as AAPB. The AAPB strains accounted for 16.67% of the total isolated bacteria; all belonged to the phylum *Pseudomonadota* and comprised five orders, seven families, and ten genera. Constructing a phylogenetic tree based on the *puf*M gene (Figure 5), we observed that AAPB strains formed five distinct orders. At the family level, the AAPB strains encompassed seven families: *Erythrobacteraceae* (34%), *Acetobacteraceae* (20%), *Azospirillaceae* (17%), *Caulobacteraceae* (12%), *Sphingomonadaceae* (7%), *Rhodobacteraceae* (5%), and *Rhizobiaceae* (5%). At the genus level, the AAPB strains comprised 10 genera: *Erythrobacter* (34%), *Skermanella* (17%), *Brevundimonas* (12%), *Belnapia* (7%), *Roseomonas* (7%), *Sphingomonas* (7%), *Fuscovulum* (5%), *Roseicella* (5%), *Pararhizobium* (3%), and *Rhizobium* (3%).

### 3.5. Exopolysaccharide Content and Sand-Fixing Ability of AAPB Strains

Based on the phenol-sulfuric acid method, the exopolysaccharide content of the purified AAPB strains was measured (Table S5). The results showed that the AAPB strain SCSIO 17546, belonging to the *Pseudomonadota*, genus *Skermanella*, exhibited the highest extracellular polysaccharide content of 0.028 ± 0.006 mg/mL. Furthermore, the evaluation of sand-binding capacity was conducted for a selected set of 11 strains with the highest extracellular polysaccharide content, as detailed in Table S6. Through rigorous ANOVA analysis, a notable divergence in coral sand aggregation thickness post-application of experimental AAPB strains compared to the control was evident (*p* < 0.05). The results showed that strains SCSIO 17432 and SCSIO 17477 exhibited remarkably robust sand-binding capabilities (*p* < 0.05); these belonged to the genera *Erythrobacter* and *Skermanella*, respectively.

## 4. Discussion

### 4.1. AAPB Community and Its Influencing Factors with Regard to Biocrust Formation

In this study, the dominant families of AAPB within the coral island’s biocrusts were *Aurantimonadaceae*, *Erythrobacteraceae*, and *Acetobacteraceae* (Figure 2A). However, contrasting these findings with prior research, the prevailing families of AAPB in desert biocrusts were *Rhodospirillaceae*, *Acetobacteraceae*, *Roseiflexaceae*, *Sphingomonadaceae*, and *Methylobacteriaceae* [13,21]. This disparity could potentially be attributed to geographical variations between the sampling sites. For instance, the dominance of *Erythrobacteraceae* might be ascribed to the coastal proximity of the coral island, where this family has been previously reported as a prevalent AAPB group in marine environments [51]. Beyond geography, the dominance of certain families might also be linked to soil characteristics. The ascendancy of *Aurantimonadaceae* and *Acetobacteraceae* could be attributed to their roles in nitrogen cycling, with the former species reported to utilize nitrate as an electron acceptor [52] and the latter associated with nitrogen fixation [53], which holds valuable implications in the nutrient-limited coral island ecosystem.

In the bare soil, the prominence of the *Porphyrobacter* genus aligns with previous findings that the taxa were often found in precursor sand materials for concrete construction [54] (Figure 2C). We speculate that such bacteria have a potential promoting effect on the formation of soil aggregates. Additionally, although the relative abundance of *Comamonadaceae* was not notably high in the bare soil, it remained significantly greater than in the biocrust samples. This observation corresponds with a study demonstrating higher prevalence of this group in nutrient-poor soil regions [55]. Notably, the prominence of the *Aurantimonadaceae* genus in coral island biocrusts resonates with prior reports [56], where a plant exhibiting resistance to invasive pathogens was found to harbor an *Aurantimonadaceae* strain. Upon genomic analysis of this strain, functionalities associated with extracellular polysaccharide synthesis, protein secretion, and biofilm formation were identified, suggesting its potential for colonization. We infer that these functionalities might also facilitate establishment within biocrusts and contribute to their formation.

Assessing the measured soil properties, TN, TP, and TOC in bare soil were all inferior to those in biocrust samples, while the pH was elevated (Table S1). The augmented levels of TN, TP, and TOC in biocrusts align with their roles in nitrogen fixation, phosphorus enhancement, and elevation of soil fertility [8,57], directly influenced by the microbial communities [4,58]. Notably, AAPB, a pivotal group in biocrust microbial communities, have been previously recognized for their potential in nitrogen fixation [23], carbon fixation [24], and extracellular polysaccharide production [25], which is essential for capturing carbon, nitrogen, and other nutrients. We speculate that the biocrust succession and extracellular polysaccharides may contain substances capable of buffering alkalinity, thus influencing soil pH [59,60].

Significant dissimilarities were observed in the AAPB community structure between bare soil and biocrusts. Our results suggested that TP, TN, TOC, pH, and Ca content significantly impacted the community structure of AAPB on the coral island (Figure 3). Similar findings have been reported in tropical coastal dune biocrusts, where cyanobacteria and green algae were prevalent, and TP and TN content influenced community structure [58]. Similarly, TOC was recognized as a critical factor influencing microbial community structure in forest soils [61]. Moreover, we ascertained a notable correlation between pH and AAPB communities. This correlation finds validation in studies demonstrating pH’s influence on bacterial and fungal communities in biocrusts across diverse regions, including the Gurbantunggut Desert (China), Intermountain West (United States), and Glacier Foreland (Norway) [60,62,63,64]. Additionally, our findings revealed a significant linkage between the AAPB communities and Ca content. While not evident in this study, it was noteworthy that Ca had been previously correlated with cyanobacterial and lichen communities in Arctic soil crusts (Dickson Land, Svalbard) and cyanobacterium-dominated biocrusts (a tropical coral island, South China Sea) [34,65]. Notably, these factors had a similar influence on AAPB communities in the Inner-Mongolian Plateau [21]. Through co-occurrence network analysis, we identified six keystone OTUs in the biocrusts, of which OTU5916 and OTU6288 belong to the *Brevundimonas* genus. This might be related to the nitrogen fixation and phosphorus solubilization functions of the *Brevundimonas* genus, which enable them to survive in extreme environments and promote the formation of biocrusts [66]. In both bare soil and biocrust samples, a considerable portion of AAPB OTUs could not be taxonomically classified at the genus or family level, indicating the presence of potentially novel or undercharacterized lineages. Notably, some of these “unclassified” taxa were identified as keystone OTUs in the co-occurrence network, suggesting that they may play essential ecological roles in the community structure and biocrust development. Due to the limitations of the reference databases and the *puf*M gene resolution, the exact phylogeny and functionality of these unclassified OTUs remain uncertain. Future studies using full-length 16S rRNA or metagenomic sequencing would be necessary to further characterize their taxonomic placement and ecological function.

### 4.2. Sand-Fixing Characteristics and Anti-Erosion Ability of AAPB Strains

Prior studies have illuminated that microorganisms within biocrusts possess the capability to synthesize polysaccharides, facilitating the agglomeration of soil particles and fortifying soil stability against erosive forces (caused by water and wind) and nutrient loss [28,67,68]. At a macroscopic level, extracellular polysaccharides manifest in granular and mucoid forms within biocrusts. The sand grains conjoin with filamentous cyanobacteria present in the early-stage biocrust, elevating soil stability [14]. Extracellular polysaccharides foster a conducive microenvironment for the accumulation of nutrients through binding. Residual substances, including extracellular enzymes, catalyze the formation of extracellular digestive systems, thus utilizing compounds as sources of nourishment and energy [69]. From a molecular perspective, disparate functional groups within extracellular polysaccharide structures offer distinct binding sites, fortifying the adhesiveness that contributes to the formation of soil aggregates [69]. The application of high-yield extracellular polysaccharide-producing microorganisms for soil structural and nutritional improvement is gaining momentum among researchers. Hence, AAPB possessing the competence to generate exopolysaccharides assume a crucial role in facilitating the formation of biocrusts. For our study, the 11 AAPB strains exhibiting elevated extracellular polysaccharide production, isolated from the tropical coral island, all displayed the ability to form soil aggregates with a certain thickness, contributing to a degree of sand fixation. Among the experimental groups, the two most proficient in sand fixation were the SCSIO 17432 (*Erythrobacter*) and SCSIO 17477 (*Skermanella*). Remarkably, SCSIO 17477 exhibited the highest extracellular polysaccharide content, aligning with prior findings that emphasized the correlation between enhanced extracellular polysaccharide production and robust sand fixation abilities [70]. Moreover, strains of the *Skermanella* genus contain genes encoding nitrogenase [71], which have the potential to fix nitrogen, while strains of the *Erythrobacter* genus have been reported to have the ability to fix carbon [72]. These characteristics may promote biocrust formation and development, especially on nutrient-poor tropical coral islands. Nonetheless, the structural integrity of these soil aggregates was relatively weak, susceptible to disruption under external forces, thus failing to establish stable structures. This might be due to insufficient exopolysaccharide content and lack of filamentous structure like filamentous cyanobacteria [68]. Although the tested AAPB strains with high EPS production and sand-binding capacity were isolated from biocrusts, it remains unclear how abundant or dominant these strains are in the native microbial community. Some genera such as *Brevundimonas* and *Skermanella* appeared both in the culture-dependent isolates and in the amplicon sequencing dataset, including as keystone OTUs, which suggests their ecological relevance. However, others with strong EPS production might be minor members or may represent strains enriched only under artificial conditions. Further quantitative analyses, such as combining FISH or metatranscriptomics with isolation, could help link functional traits (e.g., EPS production) to ecological importance in situ. In addition, while our EPS assay focused solely on AAPB isolates, future comparative studies involving non-AAPB taxa from the same environment are warranted to determine whether AAPB possess uniquely enhanced exopolysaccharide production capabilities.

These findings unequivocally showcase the pivotal contribution of the AAPB community in advancing the formation and development of biocrusts on this tropical coral island, particularly in the context of soil aggregate formation. However, we still lack sufficient understanding of the mechanism of how AAPB promote the development of the biocrusts. In follow-up studies, we will use the isolated and purified keystone species to conduct breeding experiments to explore the mechanism of AAPB affecting the development of the biocrusts.

## 5. Conclusions

We first comprehensively investigated the aerobic anoxygenic phototrophic bacteria (AAPB) composition in biocrusts on a tropical coral island in the South China Sea. In this study, biocrusts exhibited elevated total nitrogen, total phosphorus, and total organic carbon content compared to bare soil. AAPB abundance and species richness were notably higher in biocrusts compared to bare soil, aligning with qPCR results. While species diversity of AAPB was lower in biocrusts compared to bare soil, *Pseudomonadota* dominated both soil types. At the family level, *Aurantimonadaceae*, *Acetobacteraceae*, and *Rhodobacteraceae* were prominent in biocrusts, while *Erythrobacteraceae* was abundant in bare soil. In addition, a subset of AAPB operational taxonomic units could not be classified at the family or genus level. These unclassified members may represent novel or poorly characterized lineages, some of which were identified as potential keystone taxa. Their ecological roles in biocrust formation warrant further investigation using high-resolution taxonomic and functional approaches. Our findings also revealed that AAPB composition correlated with soil traits such as total nitrogen, phosphorus, organic carbon, pH, and calcium content. Co-occurrence network analysis exhibited increased stability with biocrust formation. Notably, AAPB strains showed exopolysaccharide (EPS) production potential, with strain SCSIO 17546 (*Skermanella*) demonstrating the highest EPS content. Sand-binding capacity evaluation revealed strains SCSIO 17432 (*Erythrobacter*) and SCSIO 17477 (*Skermanella*) as strong performers, highlighting their role in soil aggregation. These findings underscore the significant role of AAPB in biocrust formation, potentially impacting soil stability and nutrient retention; our findings also provide new perspectives for ecological restoration of tropical coral islands.

## Data Availability

The raw sequencing data have been deposited in the National Center for Biotechnology Information (Study accession number PRJNA1003425).

## Supplementary Materials

The following supporting information can be downloaded at: https://www.mdpi.com/article/10.3390/microorganisms13061265/s1, Figure S1. Phylogenetic tree based on species-level taxonomy of all detected OTUs in biocrust and bare soil samples. This tree provides a comprehensive overview of the phylogenetic diversity and ecological distribution of microbial communities in coral island soils. BSCs, biological soil crusts; BS, bare soil. Table S1. Alteration of the different physicochemical parameters in the biocrust and bare soil samples. Mean ± SD, and different letters indicate significant difference among group at *p* < 0.05. Table S2. Soil mechanical composition. Mean ± SD, and different letters indicate significant difference among group at *p* < 0.05. Table S3. Major topological features properties of co-occurrence networks. Table S4. Identity of the network hubs, module hubs and connectors as keystone microbes of co-occurrence networks. Table S5. Exopolysaccharide Content of AAPB Strains. Table S6. Sand-fixing ability of AAPB Strains.
