# Supplementary material for: The Community Structure of Aerobic Anoxygenic Photosynthetic Bacteria in Biocrusts on Tropical Coral Islands and Their Application in Ecological Restoration, South China Sea
Source: Microorganisms. 2025 May 29;13(6):1265. doi: 10.3390/microorganisms13061265 (PMC12195347; doi:10.3390/microorganisms13061265)
Supplement: Supplementary file 1 [file microorganisms-13-01265-s001.zip › microorganisms-3578767-supplementary.pdf]

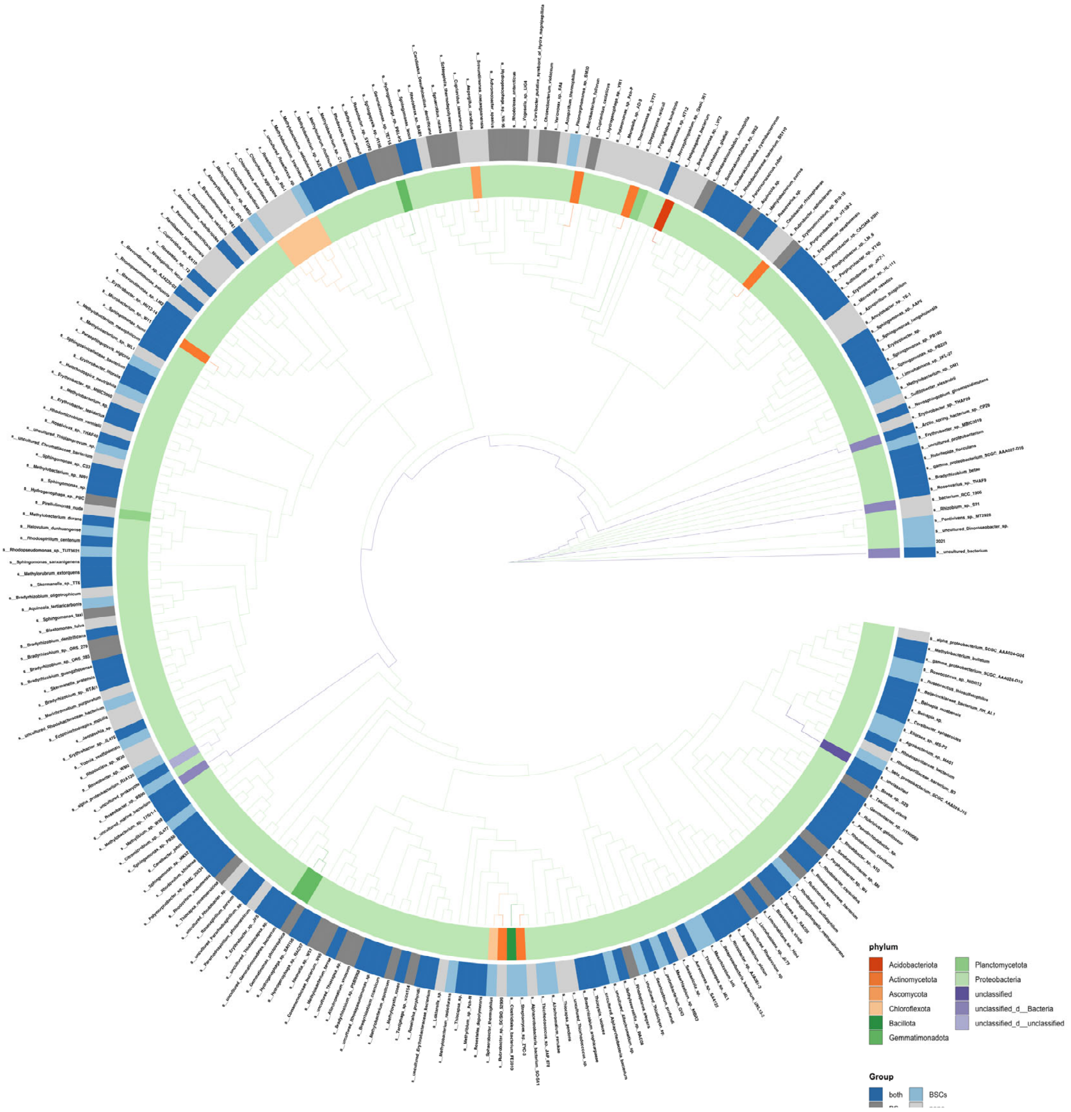

Figure S1. Phylogenetic tree based on species-level taxonomy of all detected OTUs in biocrust and bare soil samples. This tree provides a comprehensive overview of the phylogenetic diversity and ecological distribution of microbial communities in coral island soils. BSCs, biological soil crusts; BS, bare soil.

Table S1. Alteration of the different physicochemical parameters in the biocrust and bare soil samples. Mean ± SD, and different letters indicate significant difference among group at $p < 0.05$.

	Bare soil	Biocrusts
TN（g/kg）	0.68±0.03 **b**	1.95±0.06 **a**
TP（g/kg）	0.72±0.06 **b**	1.52±0.06 **a**
TOC（g/kg）	7.12±0.06 **b**	20.21±0.04 **a**
Ca（g/kg）	960.42±0.04 **a**	915.27±0.03 **b**
pH	9.46±0.21 **a**	9.12±0.16 **b**

Abbreviations: total organic carbon (TOC), total nitrogen (TN), total phosphorus (TP).

Table S2. Soil mechanical composition. Mean ± SD, and different letters indicate significant difference among group at $p < 0.05$.

	Gene copies/g
Bare soil	（2.39±0.90）$\times 10^6$ **b**
Biocrusts	（6.32±1.44）$\times 10^8$ **a**

Table S3. Major topological features properties of co-occurrence networks.

	**Bare soil**	**Biocrust**
Number of nodes	121	155
Number of edges	132	461
Positive (%)	53.03	51.41
Negative (%)	46.97	48.59
Average degree	1.091	5.948
average path length	4.859	3.959
Network diameter	14	12
Graph density	0.005	0.039
Clustering coefficient	0.177	0.339
Betweenness Centrality	40.347	132.884
modularity	0.84	0.544
Closeness Centrality	0.422	0.370

Table S4. Identity of the network hubs, module hubs and connectors as keystone microbes of co-occurrence networks.

ID	Group	Type	Class	Order	Family	Genus
OTU11998	Bare soil	Connectors	unclassified	unclassified	unclassified	unclassified
OTU5916	Biocrust	Module hubs	*Alphaproteobacteria*	*Caulobacterales*	*Caulobacteraceae*	*Brevundimonas*
OTU6054	Biocrust	Connectors	*Gammaproteobacteria*	*Chromatiales*	*Chromatiaceae*	*Thiocystis*
OTU5691	Biocrust	Connectors	*Alphaproteobacteria*	*Sphingomonadales*	*Sphingomonadaceae*	*Sphingomonas*
OTU6025	Biocrust	Connectors	unclassified	unclassified	unclassified	unclassified
OTU6288	Biocrust	Connectors	*Alphaproteobacteria*	*Caulobacterales*	*Caulobacteraceae*	*Brevundimonas*
OTU6192	Biocrust	Connectors	unclassified	unclassified	unclassified	unclassified

Table S5. Exopolysaccharide Content of AAPB Strains

Strain NO.	Taxonomy	Concentration (mg/mL)	Standard Deviation
SCSIO 17546	*Skermanella*	0.028	0.006
SCSIO 17466	*Skermanella*	0.026	0.003
SCSIO 17482	*Skermanella*	0.023	0.000
SCSIO 17432	*Erythrobacter*	0.021	0.001
SCSIO 17416	*Erythrobacter*	0.02	0.008
SCSIO 17477	*Skermanella*	0.02	0.001
SCSIO 17464	*Skermanella*	0.018	0.001
SCSIO 17440	*Erythrobacter*	0.016	0.001
SCSIO 17413	*Erythrobacter*	0.014	0.003
SCSIO 17460	*Erythrobacter*	0.014	0.000
SCSIO 17447	*Skermanella*	0.014	0.000
SCSIO 17521	*Roseicella*	0.012	0.000
SCSIO 17528	*Erythrobacter*	0.012	0.001
SCSIO 17495	*Erythrobacter*	0.011	0.001
SCSIO 17514	*Belnapia*	0.01	0.001
SCSIO 17491	*Rhizobium*	0.01	0.002
SCSIO 17530	*Brevundimonas*	0.009	0.000
SCSIO 17436	*Sphingomonas*	0.009	0.001
SCSIO 17500	*Erythrobacter*	0.009	0.000
SCSIO 17490	*Roseomonas*	0.009	0.000
SCSIO 17407	*Pararhizobium*	0.009	0.001
SCSIO 17503	*Brevundimonas*	0.009	0.001
SCSIO 17410	*Belnapia*	0.009	0.001
SCSIO 17476	*Skermanella*	0.009	0.000
SCSIO 17506	*Brevundimonas*	0.008	0.001
SCSIO 17523	*Sphingomonas*	0.008	0.001
SCSIO 17486	*Belnapia*	0.008	0.001
SCSIO 17516	*Erythrobacter*	0.008	0.000

SCSIO 17520	*Fuscovulum*	0.008	0.000
SCSIO 17522	*Brevundimonas*	0.008	0.001
SCSIO 17524	*Roseicella*	0.008	0.001
SCSIO 17493	*Roseomonas*	0.008	0.001
SCSIO 17422	*Sphingomonas*	0.008	0.001
SCSIO 17430	*Erythrobacter*	0.007	0.000
SCSIO 17509	*Erythrobacter*	0.007	0.001
SCSIO 17497	*Roseomonas*	0.007	0.001
SCSIO 17511	*Brevundimonas*	0.007	0.001
SCSIO 17518	*Fuscovulum*	0.007	0.001
SCSIO 17533	*Erythrobacter*	0.007	0.001
SCSIO 17502	*Erythrobacter*	0.007	0.001
SCSIO 17492	*Erythrobacter*	0.006	0.001
Control group		0.006	0.001

Table S6. Sand-fixing ability of AAPB Strains

**Strains NO.**	**Aggregate thickness（cm）**	**Dry sieve remainder**
SCSIO 17432	0.85±0.09	0.70±0.01
SCSIO 17413	0.84±0.12	0.47±0.05
SCSIO 17416	0.83±0.12	0.35±0.22
SCSIO 17477	0.79±0.07	0.65±0.10
SCSIO 17460	0.76±0.07	/
SCSIO 17464	0.76±0.07	/
SCSIO 17466	0.76±0.06	/
SCSIO 17482	0.72±0.10	/
SCSIO 17447	0.70±0.13	/
SCSIO 17546	0.63±0.06	/
SCSIO 17440	0.62±0.11	/
Control	0.53±0.10	0.00
